# Profile of primary childhood glaucoma at a child eye health tertiary facility in Malawi

**DOI:** 10.1186/s12886-022-02279-0

**Published:** 2022-01-31

**Authors:** Shaffi Mdala, Thokozani Zungu, Chatonda Manda, Chinsisi Namate, Elizabeth Fernando, Halima Sumayya Twabi, Gerald Msukwa, Petros Cyrus Kayange

**Affiliations:** 1 Kamuzu University of Health Sciences, Blantyre, Malawi; 2 Queen Elizabeth Central Hospital, Blantyre, Malawi; 3 Department of Mathematical Sciences, University of Malawi, Zomba, Malawi

**Keywords:** Childhood glaucoma, Intraocular pressure (IOP), Childhood blindness

## Abstract

**Background:**

To describe the clinical characteristics and treatment of primary childhood glaucoma at Queen Elizabeth Central Hospital in Blantyre, Malawi.

**Methods:**

A retrospective case notes review was undertaken of all medical records of patients aged less than 16 years with a diagnosis of primary glaucoma according to the Childhood Glaucoma Research Network Classification (CGRN) who presented from January 2016 to December 2018. The parameters extracted from the case files included age at presentation, sex, type of glaucoma, presenting complaints, laterality of ocular involvement, examination findings and the treatment modality instituted. The Mann-Whitney test was used to investigate factors associated with the intraocular pressure (IOP) in eyes that had a higher presenting IOP value compared to contralateral eyes.

**Results:**

A total of 45 subjects (80 eyes) were identified, 42 with primary congenital glaucoma (PCG) and 3 with juvenile open angle glaucoma (JOAG). The mean age for the population was 2.6 years (S.D ± 3.7) and most of the patients were male, with a male-female ratio of 2:1. The majority of patients had bilateral disease (n = 35, 77.8%) with the commonest presenting complaint being a whitish appearance of the eye (57.5%). The eyes studied had a mean IOP of 30.1 mmHg (CI 27.4–32.9), a mean horizontal corneal diameter (HCD) of 13.6 mm (CI 13.1–14.2) and a mean cup-disc-ratio `(CDR) of 0.73 (CI 0.66–0.79). In addition, 62 eyes (77.5%) had corneal haze on examination. Most patients (n = 59, 73.8%) underwent a combined trabeculotomy – trabeculectomy surgery within the study period. The median presenting IOP was significantly higher with JOAG compared to PCG (*P* = 0.02).

**Conclusion:**

PCG was the most common primary childhood glaucoma at Queen Elizabeth Central Hospital and most patients presented with bilateral eye involvement. Most of the eyes had corneal haze and JOAG was associated with a higher presenting IOP compared to PCG. Further studies to investigate the outcomes of combined trabeculotomy – trabeculectomy surgery in primary childhood glaucoma in Malawi are recommended.

**Supplementary Information:**

The online version contains supplementary material available at 10.1186/s12886-022-02279-0.

## Background

Childhood glaucoma is a group of conditions characterised by intraocular pressure (IOP) related damage to ocular structures [1]. The condition is rare in Western countries with an incidence of 1: 10,000 to 1: 70,000 and has a high incidence in Saudi Arabia, Southern India and Slovakia where it occurs in between 1:1, 250 and 1:3300 live births [2]. The relatively low incidence of childhood glaucoma and the associated decreased awareness of the condition may result in delayed detection and reduced effectiveness of treatment because of the advanced stage of disease at the point of diagnosis [3, 4]. Thus, childhood glaucoma contributes to a high percentage of blindness in children, accounting for 5% of childhood blindness worldwide with a prevalence of 18% among children in institutions for the blind [4].

With a high coverage of measles vaccination and vitamin A supplementation programmes, preventable visual loss due to vitamin A deficiency and measles have declined in Sub Saharan Africa [5]. On the other hand, childhood glaucoma and cataract are becoming increasingly important, treatable causes of childhood blindness [5, 6]. Cataract was found to be the leading cause of childhood blindness in a community-based study in Malawi with a prevalence of 35% [7]. There is a scarcity of data regarding the epidemiology and clinical characteristics of childhood glaucoma in Sub Saharan Africa and a lack of consistency in the classification of the condition [4, 5]. However, based on several reports from the region, PCG glaucoma accounts for 0.7–4% of all new glaucoma cases and it is the commonest type of childhood glaucoma [4].

Understanding the characteristics of childhood glaucoma is essential for early diagnosis, which is important in improving treatment outcomes [8]. In this regard, usage of a standardized classification system for childhood glaucoma is not only important for diagnosis and management but also improves physician communication and interpretation of research [9].

In the year 2013, an international classification system for childhood glaucoma was established by the Childhood Glaucoma Research Network (CGRN) [10]. The system provides a diagnostic guide for categorising childhood glaucoma in a systematic and reproducible manner. We could not find studies from Sub Saharan Africa reporting on the population prevalence of each subtype of childhood glaucoma according to the CGRN classification.

This study describes the profile of primary childhood glaucoma at Queen Elizabeth Central Hospital (QECH) in Blantyre, Malawi. The findings of this study add to the baseline knowledge on primary childhood glaucoma according to the CGRN classification in Sub Saharan Africa and may be valuable in planning epidemiological studies and longitudinal surveys on the management and outcomes of primary childhood glaucoma in the region.

## Methods

### Subjects

This was a hospital-based retrospective case series study that utilized data extracted from medical records of patients with primary childhood glaucoma admitted at QECH, which is the only paediatric ophthalmology referral centre in Malawi. All files of in-patients aged less than 16 years who were diagnosed with primary glaucoma on initial presentation to the facility from January 2016 to December 2018 were included in the study. Clinical records of children with glaucoma following cataract surgery or with glaucoma associated with an acquired condition such as uveitis and trauma were excluded. Files with missing information and files of patients with congenital ocular anomalies or systemic syndromes were also excluded.

The following data were ascertained from the clinical records of each subject included in the study: sex, type of glaucoma, presenting complaints, laterality of glaucoma, examination findings and the treatment modality instituted.

The study protocol adhered to the tenets of the Declaration of Helsinki for research involving human participants and was granted ethical approval by the College of Medicine Research and Ethics Committee (ethics approval certificate number P.04/19/2658). Formal informed consent was deemed to not be a requirement by the institutional review board as no personally identifiable information was collected during the review of the clinical records.

### Patient examination

Initial ophthalmic evaluation for all the patients was performed in the paediatric ophthalmology clinic at QECH. From the case notes, visual field testing was omitted and the visual acuity was not quantitatively measured at presentation in most of the patients because they could not cooperate due to young age and the presence of corneal oedema.

Other aspects of the examination were completed in the operating theatre under general anaesthesia with halothane. This included applanation tonometry which was performed using a Tonopen approximately three minutes after induction with propofol; evaluation for corneal clarity and the presence of Haab striae using a Zeiss operating miscroscope; measurement of the horizontal white to white limbal corneal diameter using a caliper and measurement of the central corneal thickness (CCT) and the axial length (AXL) using the Alcon OcuScan® RxP Measuring System. Optic disc cupping was assessed using binocular indirect ophthalmoscopy and gonioscopy was not performed due to unavailability of a Swan Jacob gonioprism at the facility.

### Classification of primary glaucoma

From the medical records of patients that fulfilled the inclusion criteria, we evaluated the clinical characteristics of each patient using the CGRN classification [10] to classify the type of primary glaucoma. In this regard, a diagnosis of PCG was assigned to children with buphthalmos in combination with at least two features from among the following signs;

- an IOP of more than 21 mmHg,
- an optic cup-disc ratio asymmetry of more than 0.2 or more in similar-sized optic discs,
- an increased AXL out of keeping with age,
- presence of Haab striae or
- a horizontal corneal diameter (HCD) of more than 11 mm in a neonate, more than 12 mm in a child under one year of age or more 13 mm at any age.

Children with at least two of these features in the absence of buphthalmos were given a diagnosis of JOAG.

PCG was further classified as being of neonatal-onset if it was detected in the first month of life, infantile-onset if it was recognised between one month of age and 2 years of age and late-recognised if it was identified after 2 years of age.

### Patient management

All children diagnosed with glaucoma at QECH are initiated on medical treatment with timolol eye drops at a twice daily dosing and they are admitted for examination under general anaesthesia. Timolol, a non-selective beta blocker, is the only anti-glaucoma topical medication available in government-run hospitals in Malawi and other classes of ocular hypotensive agents are not affordable for most patients. Surgical treatment is the definitive management offered for all children with glaucoma at the facility. In this regard, a combined trabeculotomy-trabeculectomy surgery with 5-flurouracil (5-FU) is performed. Post operatively, the patients are discharged and managed with steroid and antibiotic eye drops that are dosed frequently and tapered over 8 weeks.

### Statistical analysis

Data were captured using Microsoft Office Excel and analysed using Stata 14. Continuous numerical data were summarised as mean and standard deviation (SD) and categorical data as percentages (%). The median IOP among eyes that had a higher presenting IOP compared to contralateral eyes was computed. The Mann Whitney test was used to test whether the median IOP differed according to sex, type of primary glaucoma, onset of PCG and laterality of ocular involvement. A *P* value of <0.05 was considered to be statistically significant.

## Results

Fifty files of children with primary glaucoma were identified but five files were excluded due to missing data. Thus, 45 eligible clinical records of children with primary glaucoma were included and all patients were of African racial origin. With 31 males children and 14 female children, the male to female ratio was 2.:1. The mean age of the study population was 2.6 years (S.D ± 3.7 years) with the majority of the cases being due to PCG and over half of the patients (n = 25, 55.6%) in their first year of life. Over a third of the children with PCG had late-recognised PCG (n = 15, 35.7%). The commonest presenting complaint was a whitish appearance of the eye (n = 26, 57.8%) followed by epiphora (n = 12, 26.7%) and the majority of patients (n = 35, 77.8%) had bilateral disease, Table 1.

**Table 1:** Patient characteristics and presenting complaints

	n	%
**Age (years)**		
Mean ± SD	2.6 ± 3.7	
Range	0.1–14	
0–1 year	25	55.6
> 1 year – 3 years	9	20.0
> 3 years – 7 years	8	17.8
> 7 years – 14 years	3	6.7
**Type of glaucoma**		
PCG	42	93.3
Juvenile open angle glaucoma	3	6.7
**Subtype of PCG**		
Neonatal onset PCG	0	0
Infantile onset PCG	27	64.3
Late-recognised PCG	15	35.7
**Sex**		
Male	31	68.9
Female	14	31.1
**Presenting complaints**		
Whitish appearance	26	57.8
Epiphora	12	26.7
Reduced Vision	10	22.2
Big eyes	9	20.0
Light sensitivity	3	6.7
Other Symptoms	6	13.3
**Ocular involvement**		
Bilateral	35	77.8
Unilateral	10	22.2

*SD* standard deviation, *PCG* primary congenital glaucoma

The mean IOP of the eyes studied was 30.1 mmHg with a mean HCD of 13.6 mm and a mean AXL of 25.1 mm. Over a third of the eyes had Haab striae (n = 28, 35%) with most eyes having corneal haze (n = 62, 77.5%). With respect to management, the majority of the eyes (n = 59, 73.7%) were treated surgically and the remaining eyes (n = 21, 26.3%) were on medical management pending surgical intervention, Table 2.

**Table 2:** Clinical characteristics and management of childhood glaucoma

	n (eyes)	Mean	95% C.I
**Values of ocular parameters**			
Intraocular pressure	69	30.1 mmHg	27.4–32.9
Corneal diameter	66	13.6 mm	13.1–14.2
CCT	39	647.2um	567.1–727.4
Axial length	37	25.1 mm	23.9–26.2
Cup-to-disc ratio	58	0.73	0.66–0.79
**Corneal signs on inspection**		**Percentage of eyes**	
Haab striae	28	35%	
Corneal Haze	62	77.5%	
**Treatment modality**			
Medical only (Timolol)	21	26.3	
Surgical (CTT)	59	73.7	

*CTT* Combined trabeculotomy-trabeculectomy with 5-FU, *CCT* Central corneal thickness

The Mann-Whitney test showed that the median presenting IOP was significantly higher in patients with JOAG compared to patients with PCG (*P* = 0.02). There was no association between the presenting IOP and sex, onset of PCG or laterality of ocular involvement, Table 3.

**Table 3:** Factors associated with the presenting intraocular pressure

	IOP in the worse eye (mmHg) ^**a**^, ***n*** = 45		***P***-value ^**b**^
	Mean (SD)	Median (IQR)	
**Sex**			
Male	32.5 (11.8)	30 (21–42)	
Female	33.9 (8.8)	34 (29–38)	0.57
**Type of primary glaucoma**			
PCG	31.8 (10.3)	30 (24–38)	
JOAG	47 (8.2)	49 (38–54)	**0.02**
**Subtype of PCG,** n = 42			
Infantile onset PCG	29.6 (8.8)	29 (23–37)	
Late-recognised PCG	35.5 (11.7)	35.5 (29–45)	0.10
**Ocular involvement**			
Bilateral	34.3 (11.1)	33 (28–42)	
Unilateral	28.2 (8.9)	29 (21–34)	0.17

*IOP* Intraocular pressure, *PCG* Primary congenital glaucoma, *JOAG* Juvenile open angle glaucoma, *SD* Standard deviation

^a^ IOP in the eye with a higher IOP value than the contralateral eye

^b^ Mann-Whitney U test

## Discussion

We report the clinical presentation of primary childhood glaucoma at Malawi’s only child eye health tertiary facility. Previous research shows that PCG accounts for most of the cases of primary childhood glaucoma worldwide and it is diagnosed in the first year of life in approximately 80% of the cases [11]. This was observed in this study, where PCG accounted for 93.3% of the diagnoses of primary childhood glaucoma with 55.6% of the patients presenting in their first year of life. The findings are also comparable to a study from Northern Tanzania in which approximately 55.7% of patients with primary childhood glaucoma were aged up to one year [12]. The observation that most of the patients in the study population were male and bilateral disease was the commonest presentation is also comparable to the study from Tanzania and other reports on the presentation of PCG from Turkey and Brazil [12–14].

In the current study, infantile-onset PCG was the commonest type of PCG followed by late-recognised PCG and none of the patients had neonatal PCG. We could not find studies from Sub Saharan Africa outlining the frequency of the different subtypes of PCG, but in a study from India infantile-onset PCG was the commonest type of PCG [15]. In addition, JOAG accounted for 6.7% of the cases of primary childhood glaucoma in this study which is lower than estimates from a retrospective study in India, where the condition comprised 18.8% of all cases of primary childhood glaucoma [9].

The commonest presenting complaint in the study population was a whitish appearance of the eyes and corneal haze was present in over three-quarters of the eyes which were examined. This presentation is comparable to reports from Tanzania and Ghana where most of the children with PCG presented with severe disease and a cloudy cornea [12, 16]. The loss of corneal transparency occurs as a result of stromal oedema which results from a raised IOP and it signifies the need for urgent treatment in PCG [8]. The resultant corneal scarring and persistent opacification may have visual implications as it may lead to sensory deprivation amblyopia [8].

The mean IOP of 30.1 mmHg observed in our study is similar to the mean preoperative IOP of 28.3 mmHg (± 7.5) which was observed among 32 patients with PCG in a retrospective study across three facilities in South Eastern Nigeria [17]. It is also comparable to the mean preoperative IOP of 26.31 mmHg (±9.07) which was reported in a retrospective study from South Western Nigeria [18]. In the same study, the mean HCD at presentation was 14.04 mm (±1.38) which is also comparable to the mean HCD of 13.6 mm in our study.

The AXL of a normal eye does not exceed 18 mm at birth and it gradually increases to the adult size of 22 mm around the age of 2 years [19]. Similar to estimates from Brazil, where the children with PCG had a mean AXL of 24.57 mm, the current study reports a high mean AXL of 25.1 mm and this signifies a risk of developing axial myopia [14]. The cause for the development of high axial myopia in PCG has been considered to be the elevated intra-ocular pressure at an age in which the sclera still has plasticity [20]. Uncorrected myopia is a risk factor for developing amblyopia [21]. It is thus important for facilities managing patients with PCG to include resources for assessment and correction of refractive errors in children [8, 11].

The mean CCT of 647.2 μm in our study is higher than the mean value for paediatric populations without glaucoma [22] and is comparable to the estimate of 614.38 ± 89.41 μm reported with PCG in Brazil [14]. The increased CCT in PCG is due to corneal oedema but it may also be thinner in the absence corneal oedema due to stretching of the ocular tissues from high IOP [23]. With most patients having presented with a hazy cornea, the high mean CCT in our study population is attributable to corneal oedema.

The normal CDR is less than 0.1 in the first year of life and it can increase up to 0.3 by 15 years of age [24]. The high mean CDR of 0.73 from our study population signifies advanced disease presentation and the need for urgent surgical intervention to improve visual prognosis. The median CDR among patients with primary childhood glaucoma in Tanzania was 0.8 [12].

A diagnosis of JOAG was significantly associated with a higher presenting median IOP compared to PCG in this study. We could not find other studies that compared JOAG and PCG with respect to the presenting IOP. However, JOAG is typically known to present with high intraocular pressures. This is hypothesised to be due to an abnormal trabecular meshwork that results in reduced aqueous outflow [25].

Surgery is the preferred, definitive treatment of primary childhood glaucoma and medical therapy has a limited long-term value [26]. In our study, close to three-quarters of the eyes had undergone a combined trabeculotomy-trabeculectomy surgery. Like with other developing countries, with the majority of children with PCG presenting with a cloudy cornea, it is challenging to perform goniotomy due to poor visibility of the anterior chamber angle [27, 28]. A combined trabeculotomy–trabeculectomy (CTT) is thus usually the preferred surgical treatment and it has been reported to yield superior results in comparison with a conventional trabeculectomy [15, 16].

Apart from the small number of patients included in this study, there are some limitations which are inherent to the retrospective nature of the study. With manual documentation of medical records at the study site, we could not retrieve details regarding the clinical features and IOP measurements following surgery in most of the files. Thus, we could not study the immediate and long-term surgical outcomes or management plans of patients with primary childhood glaucoma. However, we have provided preliminary data on the clinical features and treatment of primary childhood glaucoma in Malawi which could be of value in designing future studies in Sub Saharan Africa.

## Conclusion

Most cases of primary childhood glaucoma in Malawi are due to PCG, with the infantile subtype of PCG being the commonest. Most of the patients presented with bilateral eye involvement and JOAG was associated with a higher median presenting IOP compared to PCG. Longitudinal studies to investigate the short-term and long-term treatment outcomes of primary childhood glaucoma in Malawi are recommended.

## Supplementary Information

**Additional file 1.**

## Data Availability

All data generated or analysed during this study are included in this published article (Supplementary file S1 Data. Data set).

## Abbreviations

AXL: Axial length
CCT: Combined trabeculotomy–trabeculectomy
HCD: Horizontal corneal diameter
IOP: Intraocular Pressure
JOAG: Juvenile open angle glaucoma
PCG: Primary congenital glaucoma
